# A Race Against Rupture: Saving a Diabetic From Gallbladder Perforation

**DOI:** 10.7759/cureus.97409

**Published:** 2025-11-21

**Authors:** Preethika Murugesan, Sundeep Selvamuthukumaran, B. V. Sreedevi

**Affiliations:** 1 Surgery, Mahatma Gandhi Medical College and Research Institute, Puducherry, IND; 2 General Surgery, Sree Balaji Medical College and Hospital, Chennai, IND; 3 General Surgery, SRM Medical College Hospital and Research Centre, Kanchipuram, IND

**Keywords:** biliary ascites, biliary diseases, gall bladder diseases and gallstones, gallbladder mucocele, gallbladder removal, necrotic gallbladder, type-1 gallbladder perforation

## Abstract

Gallbladder empyema represents a serious complication of acute cholecystitis and can progress to perforation, particularly in elderly or immunocompromised individuals. If not managed promptly, gallbladder perforation may lead to generalized biliary peritonitis, sepsis, and increased mortality. We report the case of a 57-year-old woman with type 2 diabetes mellitus (T2DM) who developed gallbladder empyema that progressed to free fundal perforation with associated biliary peritonitis. She was successfully treated with emergency open subtotal cholecystectomy. This case highlights the critical importance of early diagnosis and coordinated multidisciplinary intervention to prevent life-threatening outcomes.

## Introduction

Acute cholecystitis is a common clinical entity, but in some patients, especially those with comorbidities like diabetes mellitus, it may progress to empyema of the gallbladder, a suppurative condition with high risk for complications such as ischemia, necrosis, and ultimately gallbladder perforation. The incidence of gallbladder empyema in acute cholecystitis ranges from 5% to 30%, while perforation occurs in approximately 2% to 11% of cases [1,2]. Gallbladder perforation is a surgical emergency and is classified by Niemeier into three types: type I (acute free perforation with generalized peritonitis), type II (subacute localized perforation with pericholecystic abscess), and type III (chronic perforation with cholecystoenteric fistula) [3,4]. Type I, the most severe and least common, often results in biliary peritonitis, septic shock, and a high mortality rate. We report a rare case of acute free gallbladder perforation (type I) secondary to empyema in an uncontrolled diabetic patient, with generalized peritonitis requiring emergent surgical intervention. The case underscores the importance of early diagnosis, appropriate imaging, and multidisciplinary care in such critically ill patients.

## Case presentation

A 57-year-old female patient with a 10-year history of type 2 diabetes mellitus (T2DM) on oral hypoglycemics and a prior hysterectomy in July 2000, now presented to the emergency department with severe right upper quadrant abdominal pain and high-grade fever for five days, accompanied by nausea and loss of appetite for two days. On arrival, she was toxic-appearing, febrile, tachycardic, tachypneic, and hypotensive. Clinical icterus was noted. Abdominal examination revealed tenderness in the right hypochondrium and epigastrium initially without guarding or rigidity. Baseline laboratory investigations are summarized in Table 1. Initial ultrasound abdomen demonstrated acute calculus cholecystitis with associated bilateral pleural effusion. T

Postoperatively, the patient required intensive care unit (ICU) management for septic shock, diabetic ketoacidosis (DKA), and acute respiratory distress syndrome (ARDS), necessitating mechanical ventilator support. Glycemic control was maintained with continuous insulin infusion, and broad-spectrum intravenous antibiotics were continued. With aggressive resuscitative measures, including sedation, fluid correction, ventilatory support, and chest physiotherapy, the patient showed steady clinical improvement. She was extubated on postoperative day (POD) 3, the subhepatic drain was removed on POD 7 after minimal output, and she was discharged in stable condition on POD 10.

At two-week follow-up, the surgical wound had healed well with no bile leak or residual collection on ultrasound. At one-month follow-up, she remained asymptomatic with good glycemic control and no recurrence of abdominal pain or jaundice. Histopathological examination revealed multiple gallstones with a congested, thickened wall (0.7-0.8 cm) showing features of acute-on-chronic cholecystitis, confirming the diagnosis (Figure 4).

**Figure 1:**
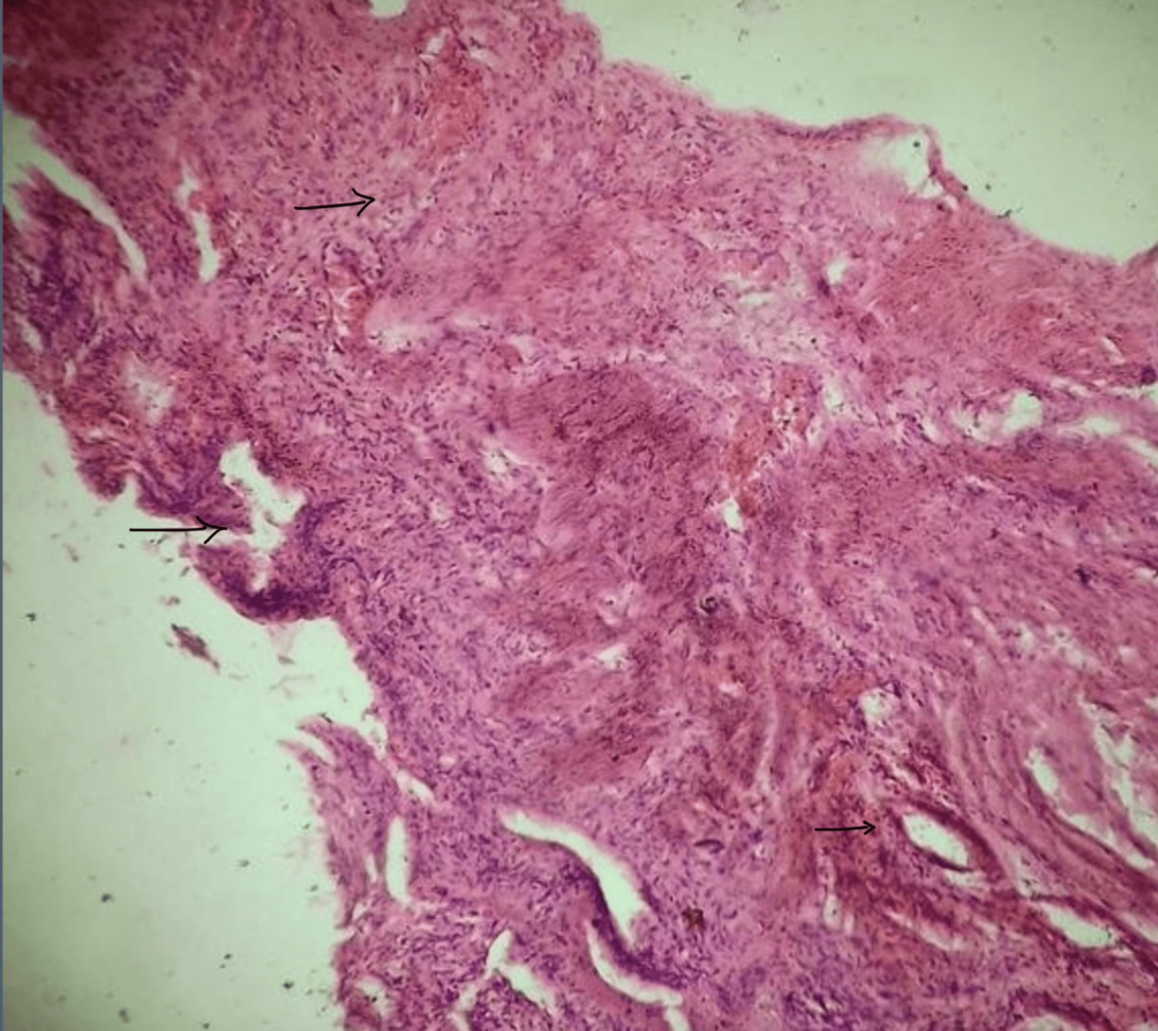
Gallbladder tissue section showing mucosal ulceration with Rokitansky-Aschoff sinuses and fibrosis GB wall GB: gallbladder

## Discussion

Gallbladder perforation (GBP) represents a rare but potentially fatal complication of acute cholecystitis, with incidence estimates ranging from approximately 2% to 11% of acute cholecystitis cases. It occurs when inflammation progresses to transmural necrosis, leading to rupture of the gallbladder wall [1,2]. This condition is more frequently observed in patients with delayed diagnosis or in those with underlying comorbidities that compromise immunity or vascular perfusion, such as diabetes mellitus, cardiovascular disease, or advanced age [3,4]. The current case highlights a classic example of type I (acute free) GBP, which presents with generalized peritonitis and sepsis, demanding urgent surgical management [3,5].

The pathophysiology of free GBP begins with obstruction of the cystic duct, typically by a gallstone, leading to increased intraluminal pressure, bile stasis, and inflammation [1,2]. In severe cases, the combination of ischemia and bacterial infection results in suppuration and necrosis of the gallbladder wall [2]. The fundus of the gallbladder is the most common site of perforation due to its relatively poor blood supply compared to other parts of the organ [2]. This was corroborated in our case, where the fundal region was the site of perforation. Diabetes mellitus, a key comorbidity in our patient, contributes significantly to this progression due to its adverse effects on neutrophil function, microvascular perfusion, and wound healing [4,5].

Patients with GBP often present with nonspecific symptoms such as fever, abdominal pain, and signs of systemic toxicity. In many instances, perforation is only discovered intraoperatively [5,6]. The initial presentation of our patient, right upper quadrant pain, fever, and other signs, was initially attributed to another cause (DKA and presumed urinary infection). This highlights a common diagnostic challenge: when multiple systems are involved, the classical triad of fever, right upper quadrant pain, and leukocytosis may not always clearly point to perforation [7,8].

Imaging studies are crucial. Ultrasound, though widely available, may be limited by operator dependency and bowel gas; it often identifies gallstones, gallbladder wall thickening, and pericholecystic fluid but has a sensitivity of only 30-70% for detecting perforation [1,9]. In contrast, contrast-enhanced computed tomography (CECT) of the abdomen provides higher sensitivity and specificity for identifying features such as intraperitoneal bile, wall defects, and localized collections [10,2]. In our case, the initial ultrasound suggested cholelithiasis with empyema but failed to detect perforation; it was only upon clinical deterioration that CECT revealed the free fundal perforation, subcapsular liver collection, and diffuse peritoneal involvement, prompting emergency surgery.

Surgery remains the definitive treatment for GBP [2,6,7]. While laparoscopic cholecystectomy is the gold standard for uncomplicated acute cholecystitis, it is often not feasible in cases of perforation, dense adhesions, or unstable hemodynamics. In such situations, open cholecystectomy is preferred. In this case, due to frozen Calot’s triangle and dense pericholecystic adhesions, a fundus-first open cholecystectomy was performed [6,10]. Some studies also suggest that timely percutaneous interventions or drainage may act as a bridge in high-risk patients, but definitive surgery remains the mainstay.

The prognosis of free GBP depends on early recognition, timely surgical intervention, and effective postoperative care [2,3,5]. Mortality in untreated or delayed cases may exceed 30-50%, especially in elderly or immunocompromised individuals. Prompt surgery can reduce mortality to less than 10% in selected series [2,3]. In a large series, morbidity was 57.7% and mortality 9.5% in patients with GBP, with preoperative sepsis being the only independent predictor of mortality. PubMed preventive strategies include early elective cholecystectomy in patients with symptomatic gallstones, education about recognition of acute cholecystitis symptoms, and regular monitoring and control of blood glucose levels to reduce complications [4,6].

This case reinforces the importance of early intervention in gallstone disease, particularly in high-risk populations. Delayed management increases the risk of gallbladder gangrene, empyema, perforation, and septic complications, and early surgical intervention in high-risk patients is both life-saving and essential to optimal outcomes [2,5,6]. The incorporation of structured diagnostic pathways (e.g., early CT in suspected cholecystitis with comorbidities) and risk stratification may further improve outcomes [11].

## Conclusions

This case highlights the importance of high clinical suspicion and prompt imaging in diagnosing free gallbladder perforation, especially in high-risk populations such as diabetics. Free perforation of the gallbladder with biliary peritonitis is a surgical emergency with significant morbidity and mortality if not treated in time. Early diagnosis, aggressive resuscitation, timely surgical intervention, and comprehensive multidisciplinary care are critical to achieving favorable outcomes in such patients. Subtotal cholecystectomy is a valuable surgical strategy in the presence of severe inflammation and frozen anatomy.
